# Supplementary material for: MetaQTL: a package of new computational methods for the meta-analysis of QTL mapping experiments
Source: BMC Bioinformatics. 2007 Feb 8;8:49. doi: 10.1186/1471-2105-8-49 (PMC1808479; doi:10.1186/1471-2105-8-49)
Supplement: Additional File 7 — MetaQTL Package : jar file and tutorial. This Zip archive contains both the MetaQTL JAR file and the files of the tutorial. [file 1471-2105-8-49-S7.zip › org.inra.metaqtl/doc/org/metaqtl/numrec/NumericalUtilities.html]

NumericalUtilities


|  |  |  |
| --- | --- | --- |
| **Overview** \| **Package** \| **Class** \| **Use** \| **Tree** \| **Deprecated** \| **Index** \| **Help** | |  |
| PREV CLASS   **NEXT CLASS** | **FRAMES**    **NO FRAMES**     **All Classes** |  |
| SUMMARY: NESTED \| FIELD \| CONSTR \| METHOD | DETAIL: FIELD \| CONSTR \| METHOD |  |


---


## org.metaqtl.numrec Class NumericalUtilities

```
java.lang.Object
  org.metaqtl.numrec.NumericalUtilities
```

---

``` public final class NumericalUtilities extends java.lang.Object ```

Class Description Here

**Author:**
:   Jean-Baptiste Veyrieras

---

| **Nested Class Summary** | |
| --- | --- |
| `static interface` | `NumericalUtilities.Function` |
| `static interface` | `NumericalUtilities.NRFunction` |


| **Field Summary** | |
| --- | --- |
| `static double[]` | `GAMCOF` |


**Constructor Summary**

`NumericalUtilities()`


| **Method Summary** | |
| --- | --- |
| `static double` | `beta(double z, double w)`             Returns the beta function I(a,b) |
| `static double` | `betacf(double a, double b, double x)`             Used by betai : Evaluates continued fraction for incomplete beta function by modified Lentz's method. |
| `static double` | `betai(double a, double b, double x)`             Returns the incomplete beta function Ix(a,b) |
| `static double` | `ci2sd(double ci)` |
| `static double` | `dfridr(NumericalUtilities.Function f, double x, double h)`             Returns the derivative of a function func at a point x by Ridders' method of polynomial extrapolation. |
| `static double` | `DMAX(double a, double b)`             Returns the max between `a` and `b` |
| `static void` | `DSWAP(double[] a, int i, int j)`             Swaps elements i and j in a. |
| `static double` | `gammln(double xx)` |
| `static double` | `gammp(double a, double x)` |
| `static double` | `gauss(double x)`             This routine returns the value at x of the normal centered gaussian density. |
| `static double[]` | `gcf(double a, double x)` |
| `static double[]` | `gser(double a, double x)` |
| `static int` | `IMIN(int a, int b)`             Returns the min between `a` and `b` |
| `static void` | `indexx(int n, double[] arrin, int[] indx)`             Sort the given array of double arrin[1..n] without destroying it and returns the sorted indices of the array elements into indx[1..n]. |
| `static void` | `ISWAP(int[] a, int i, int j)`             Swaps elements i and j in a. |
| `static double` | `PYTHAG(double a, double b)`             Computes sqrt(a^2+b^2) |
| `static double` | `rtsafe(NumericalUtilities.NRFunction funcd, double x1, double x2, double xacc)` |
| `static double` | `sd2ci(double sd)` |
| `static double` | `SIGN(double a, double b)`             Returns abs(a) if b > 0, -abs(a) otherwise. |
| `static double` | `SQR(double a)`             Returns a^2 |

**Methods inherited from class java.lang.Object**

`clone, equals, finalize, getClass, hashCode, notify, notifyAll, toString, wait, wait, wait`

**Field Detail**

### GAMCOF

```
public static double[] GAMCOF
```


**Constructor Detail**

### NumericalUtilities

```
public NumericalUtilities()
```


**Method Detail**

### SIGN

```
public static double SIGN(double a,
                          double b)
```

:   Returns abs(a) if b > 0, -abs(a) otherwise.

    :   **Parameters:**: `a` -: `b` - **Returns:**: +/- abs(a)

---


### DMAX

```
public static double DMAX(double a,
                          double b)
```

:   Returns the max between `a` and `b`

    :   **Parameters:**: `a` -: `b` - **Returns:**: max(a,b)

---


### IMIN

```
public static int IMIN(int a,
                       int b)
```

:   Returns the min between `a` and `b`

    :   **Parameters:**: `a` -: `b` - **Returns:**: min(a,b)

---


### PYTHAG

```
public static double PYTHAG(double a,
                            double b)
```

:   Computes sqrt(a^2+b^2)

    :   **Parameters:**: `a` -: `b` - **Returns:**: sqrt(a^2+b^2) the hypothenus

---


### SQR

```
public static double SQR(double a)
```

:   Returns a^2

    :   **Parameters:**: `a` - **Returns:**: a^2

---


### gauss

```
public static double gauss(double x)
```

:   This routine returns the value at x of the normal
    centered gaussian density.

---


### sd2ci

```
public static double sd2ci(double sd)
```

:   **Parameters:**: `sd` - **Returns:**

---


### ci2sd

```
public static double ci2sd(double ci)
```

:   **Parameters:**: `ci` - **Returns:**

---


### gammln

```
public static double gammln(double xx)
```

---


### gammp

```
public static double gammp(double a,
                           double x)
```

---


### gcf

```
public static double[] gcf(double a,
                           double x)
```

---


### gser

```
public static double[] gser(double a,
                            double x)
```

---


### betacf

```
public static double betacf(double a,
                            double b,
                            double x)
```

:   Used by betai : Evaluates continued fraction for incomplete beta function
    by modified Lentz's method.

    :   **Parameters:**: `a` -: `b` -: `x` - **Returns:**

---


### betai

```
public static double betai(double a,
                           double b,
                           double x)
```

:   Returns the incomplete beta function Ix(a,b)

    :   **Parameters:**: `a` -: `b` -: `x` - **Returns:**

---


### beta

```
public static double beta(double z,
                          double w)
```

:   Returns the beta function I(a,b)

    :   **Parameters:**: `z` -: `w` - **Returns:**

---


### indexx

```
public static void indexx(int n,
                          double[] arrin,
                          int[] indx)
```

:   Sort the given array of double arrin[1..n] without destroying it and
    returns the sorted indices of the array elements into indx[1..n].

    :   **Parameters:**: `n` - the array size: `arrin` -: `indx` -

---


### rtsafe

```
public static double rtsafe(NumericalUtilities.NRFunction funcd,
                            double x1,
                            double x2,
                            double xacc)
```

:   **Parameters:**: `function` -: `x1` -: `x2` -: `xacc` - **Returns:**

---


### dfridr

```
public static double dfridr(NumericalUtilities.Function f,
                            double x,
                            double h)
```

:   Returns the derivative of a function func at a point x by Ridders' method of
    polynomial extrapolation. The value h is input as an extimated initial stepsize;
    it need not to be small, but rather should an increment in x over which func changes
    substantially. The error is returned and the derivative is set in function f.

---


### ISWAP

```
public static void ISWAP(int[] a,
                         int i,
                         int j)
```

:   Swaps elements i and j in a.

    :   **Parameters:**: `a` -: `i` -: `j` -

---


### DSWAP

```
public static void DSWAP(double[] a,
                         int i,
                         int j)
```

:   Swaps elements i and j in a.

    :   **Parameters:**: `a` -: `i` -: `j` -


---


|  |  |  |
| --- | --- | --- |
| **Overview** \| **Package** \| **Class** \| **Use** \| **Tree** \| **Deprecated** \| **Index** \| **Help** | |  |
| PREV CLASS   **NEXT CLASS** | **FRAMES**    **NO FRAMES**     **All Classes** |  |
| SUMMARY: NESTED \| FIELD \| CONSTR \| METHOD | DETAIL: FIELD \| CONSTR \| METHOD |  |


---
